# The Therapeutic Mechanisms of Honey in Mitigating Toxicity from Anticancer Chemotherapy Toxicity: A Review

**DOI:** 10.3390/jox14030063

**Published:** 2024-08-20

**Authors:** Debalina Bose, Ademola C. Famurewa, Aman Akash, Eman M. Othman

**Affiliations:** 1 P.K. Sinha Centre for Bioenergy and Renewables, Advanced Technology Development Centre, Indian Institute of Technology, Kharagpur 721302, West Bengal, India; debalinabose22@gmail.com; 2 Department of Medical Biochemistry, Faculty of Basic Medical Sciences, Alex Ekwueme Federal University, Ndufu-Alike Ikwo, P.M.B. 1010, Abakaliki 482131, Nigeria; 3 Centre for Natural Products Discovery, School of Pharmacy and Biomolecular Sciences, Faculty of Science, Liverpool John Moores University, Byrom Street, Liverpool L3 3AF, UK; 4 Department of Bioinformatics, Biocenter, University of Wuerzburg, Am Hubland, 97074 Wuerzburg, Germany; aman.akash@stud-mail.uni-wuerzburg.de; 5 Department of Biochemistry, Faculty of Pharmacy, Minia University, Minia 61519, Egypt; 6 Cancer Therapy Research Center (CTRC), Department of Biochemistry-I, Biocenter, University of Wuerzburg, Theodor-Boveri-Weg 1, 97074 Wuerzburg, Germany

**Keywords:** chemotherapy toxicity, adjuvant therapy, mitigation, honey

## Abstract

Within the domain of conventional oncochemotherapeutics, anticancer chemotherapy (AC) has emerged as a potent strategy for the treatment of cancers. AC is the mainstay strategy for solid and non-solid cancer treatment. Its mechanistic action targets the blockage of DNA transcription and the dysregulation of cell cycle machinery in cancer cells, leading to the activation of death pathways. However, the attendant side effect of toxicity inflicted by AC on healthy tissues presents a formidable challenge. The crucial culprit in the AC side effect of toxicity is unknown, although oxidative stress, mitochondrial impairment, inflammatory cascades, autophagy dysregulation, apoptosis, and certain aberrant signaling have been implicated. Honey is a natural bee product with significant health benefits and pharmacological properties. Interestingly, the literature reports that honey may proffer a protection mechanism for delicate tissue/organs against the side effect of toxicity from AC. Thus, this review delves into the prospective role of honey as an alleviator of the AC side effect of toxicity; it provides an elucidation of the mechanisms of AC toxicity and honey’s molecular mechanisms of mitigation. The review endeavors to unravel the specific molecular cascades by which honey orchestrates its mitigating effects, with the overarching objective of refining its application as an adjuvant natural product. Honey supplementation prevents AC toxicity via the inhibition of oxidative stress, NF-κB-mediated inflammation, and caspase-dependent apoptosis cascades. Although there is a need for increased mechanistic studies, honey is a natural product that could mitigate the various toxicities induced by AC.

## 1. Introduction

In the realm of cancer treatment, AC stands out as a potent and widely employed strategy against malignant tumors. While this therapeutic intervention targets cancerous cells, the collateral damage inflicted on healthy tissues often results in unpalatable adverse effects known as AC toxicity [1]. AC-induced toxicity impacts patients’ well-being and therefore constitutes a source of worry for oncologists. There are pieces of evidence that toxicity affects the healthy liver, kidney, brain, placenta, ovary, testis, heart, lungs, spleen, bladder, and intestine [2,3,4,5]. The literature strongly implicates oxidative stress, apoptosis, autophagy, and inflammation as the dominant pathways that trigger the AC side effect of toxicity [2,3,6]. Accumulating findings also implicate a delicate adverse role of ferroptosis in the development of AC-induced nephrotoxicity and cardiotoxicity [7,8,9].

The recognition of the need for adjunctive approaches to mitigate these unpalatable side effects has compelled researchers to turn to natural products with the potential to mitigate toxicity. More importantly, natural products with the potential to combat oxidative stress and inflammation are target agents for adjuvant combination therapy. Among them, honey has emerged as a promising candidate due to its multifaceted pharmacological activities [10]. Honey is a natural product and functional food with several appealing health benefits and pharmacological effects, making it an intriguing subject of study for its potential to alleviate AC-induced toxicity. In various studies, honey and its bioactive components have demonstrated the capacity to combat the ROS-mediated induction of oxidative inflammation and apoptosis [11,12,13,14]. Moreover, managing AC-related side effects and toxicities requires close monitoring, supportive care, and adjustments to treatment regimens, aiming to optimize outcomes for patients with cancer. Advances in medical research continue to explore ways to minimize AC toxicity and enhance the overall safety and efficacy of cancer treatment.

This review explores the intricate dynamics between honey and the toxicity side effect linked to AC and provides valuable insights into the fundamental molecular mechanisms of mitigation by honey. We searched relevant databases, including Web of Science, Scopus, PubMed, and Google Scholar, for published papers on AC-induced toxicity, the amelioration of chemotherapy toxicity by honey from different countries, and the protective mechanisms of honey against antineoplastic drugs. By gaining a comprehensive understanding of these molecular underpinnings, researchers strive to optimize the application of honey as an adjuvant therapy alongside AC treatments. The ultimate goal is to augment the overall effectiveness of cancer management while concurrently minimizing associated toxicities. The significance of these insights extends beyond the realm of advancing cancer therapeutics; it also holds promise for enhancing the quality of life for patients during and after treatment. Navigating the complex landscape of cancer research, the potential of honey as a mitigator against AC toxicity emerges as a beacon of hope in the pursuit of cancer care that is not only more effective but also more patient friendly.

## 2. Mechanism of Anticancer Chemotherapy Toxicity

AC, a frequently employed therapeutic approach for cancer treatment, encompasses the utilization of chemical drugs to eliminate or hinder the proliferation of swiftly dividing cancer cells. Although AC proves efficacious in addressing cancer cells, it also impacts ordinarily functioning, rapidly dividing healthy cells, resulting in a spectrum of side effects and toxicities. The clinical symptoms of AC side effects include nausea, vomiting, fatigue, weakness, hair loss, compromised immune function, anemia, neutropenia, and thrombocytopenia [15,16,17]. AC drugs form a varied category of chemical agents specifically crafted to combat and impede the proliferation of cancer cells by targeting different stages of the cancer cell cycle, disrupting processes of cell division, DNA replication, and protein synthesis [2,18].

**Figure 1:**
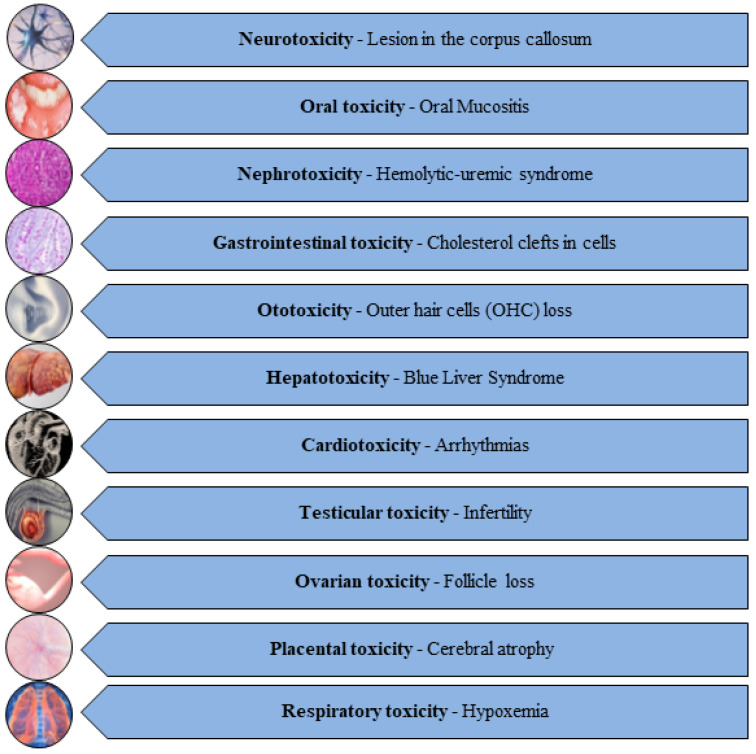
Diverse range of toxicities induced by AC drugs [22,23,24,25,26,27,28,29,30,31,32].

Unfortunately, these potent AC drugs (Table 1) exert detrimental effects on vital organs like the liver, kidneys, ovary, testes, placenta, brain, spinal cord, spleen, heart, lungs, bladder, and bone marrow, giving rise to hepatotoxicity, nephrotoxicity, testiculotoxicity, neurotoxicity, cardiotoxicity, and ovarian, pulmonary, and placental toxicities [19,20,21] (Figure 1).

Although the crucial culprit of AC toxicity is still unknown, a robust body of evidence shows that AC drugs are triggers of reactive oxygen species (ROS) implicated in impaired antioxidant homeostasis in healthy organs [6,33]. AC drug exposure suppresses the tissue activities of antioxidant enzymes and levels of non-enzymatic antioxidant apparatus. In preclinical studies, AC drugs such as doxorubicin, methotrexate, cisplatin, cyclophosphamide, docetaxel, 5-fluorouracil, etc., have suppressed antioxidant homeostasis in various healthy organs, leading to redox imbalance and oxidative stress [34]. Oxidative stress has been recognized as the chief mechanism provoking AC toxicity.

Studies indicate that the AC-mediated excess ROS in non-cancer organs results in an oxidative stress status which may impair mitochondrial function and DNA replication and trigger the aberrant modulation of apoptosis and inflammation, including signaling pathways such as Nrf2/Keap-1/HO-1, Akt/mTOR/GSK-3β, NF-κB/p38/MAPK, and NLRP3 inflammasome [7,34,35]. Moreover, recently, the involvement of ferroptosis has been reported in the AC toxicity process. Ferroptosis is a non-apoptotic programmed cell death orchestrated by iron accumulation, the iron-dependent production of lipid peroxidation, the enhancement of iron-catalyzing Fenton reaction, and reduced glutathione (GSH) level and GSH peroxidase-4 (GPx4) activity [8,36].

**Table 1:** Different classes of antineoplastic drugs.

Anticancer Agents		
Type	Drug	References
**Alkylating Agents**	Cisplatin	[37]
	Temozolomide	[38]
	Busulfan	[39]
	Ifosfamide	[40]
	Cyclophosphamide	[41]
**Protein Kinase Inhibitors**	Duvelisib	[42]
	Crizotinib	[43]
	Lenvatinib	[44]
	Olaparib	[45]
**Monoclonal Antibodies**	Necitumumab	[46]
	Rituximab	[47]
	Durvalumab	[48]
	Moxetumomab	[49]
**Antibiotics**	Bleomycin	[50]
	Idarubicin	[51]
	Valrubicin	[52]
	Daunorubicin	[53]
	Doxorubicin	[7]
**Vinca Alkaloids**	Vinblastine	[54]
	Vincristine	[55]
	Vinorelbine	[56]
**Hormonal Agents**	Flutamide	[57]
	Cyproterone	[58]
	Triptorelin	[59]
	Tamoxifen	[60]
**Antimetabolites**	Methotrexate	[61]
	Fluorouracil	[62]
	Mercaptopurine	[63]
	Azacitidine	[64]
**Taxanes**	Paclitaxel	[65]
	Docetaxel	[65]
	Cabazitaxel	[65]
**Others**	Omacetaxine	[66]
	Tazemetostat	[67]
	Lenalidomide	[68]
	Thalidomide	[69]

The induction of lipid peroxidation and suppression of GPx-4 in the ferroptosis mechanism cause ROS-mediated tissue injury during exposure to AC in various animal models [7,9]. Studies on ferroptosis so far have shown that it is different from necrosis, autophagy, and apoptosis, and its induction has been shown to reverse chemo resistance and potentiate cancer treatment [70,71]. However, its aberrant role in the potentiation of the AC side effect of organ toxicity is a prevailing dilemma.

## 3. Honey: A Natural Food Product

Beyond its traditional use, honey plays a multifaceted role in health and well-being. In Ayurveda, known as “Madhu” in Sanskrit, honey holds a revered status, embodying a healing tradition with its “sattvic” qualities that balance overall health [72,73]. Modern honey-infused products offer diverse health benefits, acting as a nutraceutical powerhouse with instant energy from natural sugars, catering to athletes and quick revitalization [74]. Clinical studies underscore honey’s therapeutic properties, including varieties like Manuka honey, which exhibits potential in various health applications ranging from allergy relief and wound care to catheter-associated infections in patients undergoing hemodialysis, and it even has preventive measures against cervical cancer and chemotherapy-induced toxicity [75,76,77,78,79,80]. Moreover, honey extends its significance beyond alternative medicine, playing a vital role in cuisine and beauty. Whether used as a sugar substitute in culinary delights or as a key ingredient in skincare products, honey remains a symbol of the wonders of nature, offering a delightful and healthful addition to both culinary and wellness pursuits [81,82,83].

### 3.1. Geographical Heterogeneity of Honey

Honey exhibits a rich tapestry of flavors and characteristics across various countries owing to the distinct floral sources and geographical environments that influence its exquisite profile and production. In Greece, for instance, thyme honey is celebrated for its robust flavor and aromatic notes, reflective of the country’s abundant thyme fields [84]. New Zealand is renowned for its Manuka honey, prized for its unique antibacterial properties derived from the Manuka tree [85]. Acacia honey from Hungary is cherished for its light color and delicate taste, sourced from the nectar of the acacia flower [86]. In Turkey (a top global honey producer), a wealth of honey varieties such as chestnut, pine, and flower honey flourish [87]. Spain showcases its high-quality honey, including the prized lavender and rosemary varieties, often relocating hives to diverse floral sources [88]. Mexico boasts a rich beekeeping tradition, crafting unique honey flavors, with the Yucatan Peninsula being renowned for Melipona beekeeping [89]. Argentine honey is lauded for its purity, featuring eucalyptus and Chilca varieties [90]. In China (one of the world’s largest honey producers), a plethora of types like jujube honey thrive [91]. Australia not only offers Manuka honey but also an array of options like eucalyptus, macadamia, and leatherwood honey [92]. Honey with distinct flavors, like lavender honey, is produced in France [93], while India’s rich beekeeping heritage yields multifloral and lychee honey [94]. These global honey practices celebrate the versatility and cultural richness of apiculture, delivering a delightful selection of honey for diverse palates and culinary experiences worldwide. The bioactivities of honey influenced by their botanical and geographical origin are depicted in Table 2.

### 3.2. Natural Compounds in Honey and Their Bioactivity

Honey’s diverse health benefits stem from its rich composition, including sugars, phenolics, flavonoids, proteins, enzymes, organic acids, amino acids, vitamins, pollens, and minerals. Water, glucose, and fructose contribute to its preservative property, sweetness, and energy [95,96]. Trace elements, vitamins (C and B), and minerals (calcium, iron, and magnesium) support tissue regeneration [97,98]. Pollen grains enhance its therapeutic potential [99], while amino acids boost its nutritional value [100]. Enzymes like invertase, catalase, and glucose oxidase provide antimicrobial properties [101]. Phenolic compounds and flavonoids functioning as antioxidant agents play a pivotal role in counteracting detrimental free radicals, thereby contributing to the potential health benefits associated with honey [102]. Organic acids, including acetic acid, citric acid, and formic acid, contribute to the acidic nature and flavor profile of honey [103]. The combination of these bioactive compounds gives honey its multifaceted qualities, with anti-inflammatory, antioxidant, and antimicrobial attributes. This versatility positions honey as a natural product with potential benefits for wound healing, immune support, and overall health.

**Figure 2:**
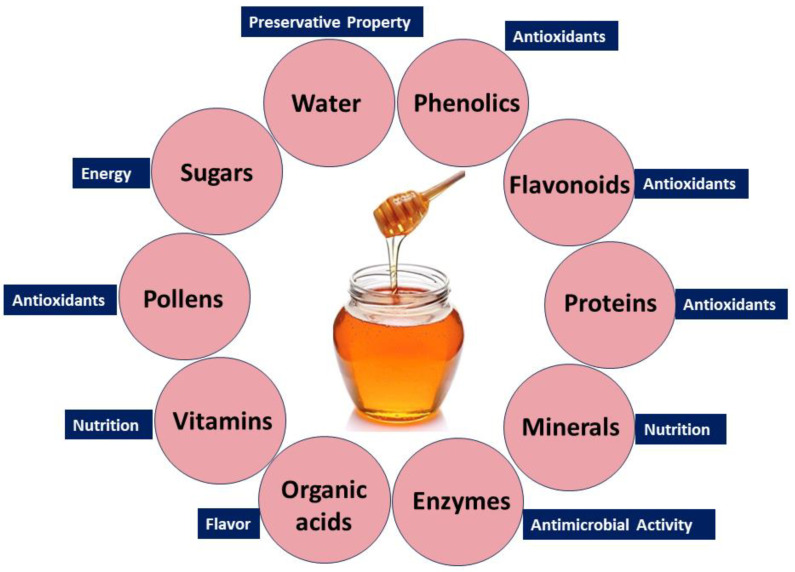
Bioactive compounds in natural honey and their biological benefits.

Nevertheless, it is crucial to acknowledge that the makeup and bioactivity of honey may differ depending on the botanical origin and geographic location. Figure 2 depicts the main constituents present in honey.

### 3.3. Honey and Its Pharmacological Properties

Honey’s pharmacological prowess, inhibiting bacterial and fungal growth, is complemented by the osmotic effect and acidic pH, contributing to its preservation quality [88,104,105,106]. This antimicrobial and antioxidant-rich nature positions honey as an ally in managing chronic inflammation and provides analgesic effects, fostering tissue regeneration [107,108,109]. Honey’s multifaceted analgesic effects showcase its versatility in traditional and complementary medicine, impacting pain perception [101,110] and wound healing [111]. Honey’s hypoglycemic and hypolipidemic effects further contribute to managing diabetes and hyperlipidemia, offering potential benefits for cardiovascular health [112,113,114].

**Figure 3:**
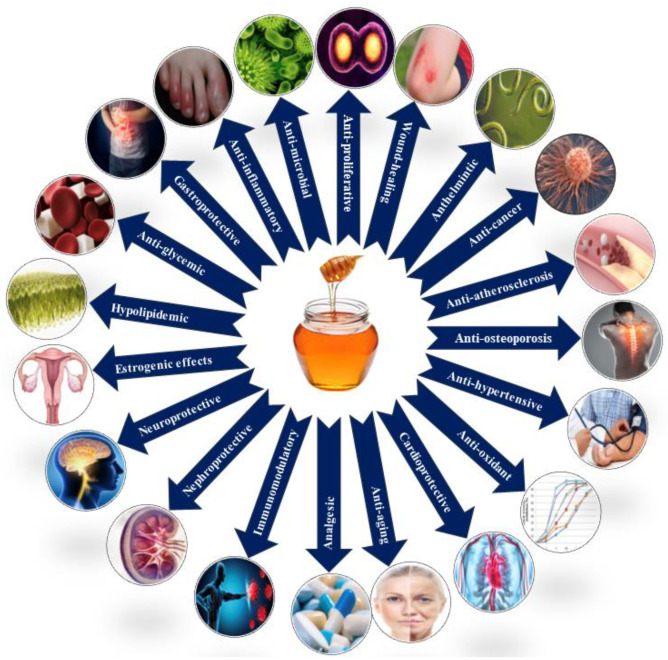
Pharmacological properties of honey.

The antioxidant attributes of honey promote a favorable lipid profile and may influence genes associated with lipid metabolism, enhancing the body’s ability to regulate lipid levels [114,115]. Honey’s anti-hypertensive property and immunomodulatory effect contribute to cardiovascular health, showcasing its potential in regulating inflammatory processes linked to elevated lipid levels [116,117,118,119]. Recent research indicates that honey’s potential anti-cancer properties include the inhibition of cancer cell growth, immune responses, and the arrest of metastatic processes [112,120,121,122,123]. Honey’s neuroprotective effects extend to the nervous system, offering advantages for brain health, including the potential regulation of neurotransmitter levels and the prevention of neurodegenerative diseases [124,125,126,127]. Figure 3 shows the therapeutic activity of honey.

Studies suggest that honey may have nephroprotective effects, supporting optimal kidney function and potentially exhibiting diuretic effects [14,128,129]. Its estrogenic characteristics may contribute to hormonal modulation, offering potential benefits for conditions related to hormonal balance, including menopausal symptoms [130,131,132]. Honey’s potential anti-helminthic or anti-parasitic activity is linked to its high sugar content and slightly acidic pH, making it inhospitable for parasitic organisms [133,134]. In traditional medicine, honey’s antimicrobial activity, anti-inflammatory features, and ability to stimulate tissue regeneration make it a staple for wound healing, including gastrointestinal issues [135,136,137].

Honey’s anti-proliferative, anti-aging, and anti-atherosclerotic qualities make it a preventive healthcare agent, impacting cancer research and skin health [82,112,138]. Emerging research explores honey’s potential in preventing atherosclerosis through its polyphenols and flavonoids, as well as its positive impact on lipid metabolism [139,140]. Continuous research aims to uncover the specific mechanisms behind honey’s positive effects on bone health [141]. Table 2 depicts the therapeutic activities of honey based on its bioactive constituents.

**Table 2:** Honey: its botanical and geographical origin, active constituents, and bioactivities.

Honey	Origin	Components	Bioactivity	Reference
Tualang honey	Malaysia	Neurotransmitters (choline and acetylcholine)	Anti-depressant	[142]
Thyme honey	Greece	H_2_O_2_ activity and low acidity	Anti-microbial	[143]
Sidr honey	Kingdom of Saudi Arabia and Pakistan	Polyphenols (caffeic acid and its phenyl esters)	Anticancer	[144]
Heather honey	Romania	Phenolics	Antibacterial	[145]
Avocado honey	Spain	*o*-vanillin, ellagic acid, ferulic acid, and hydroxycinnamic acid	Anti-Alzheimer’s	[146]
Buckwheat honey	United States	Sugar	Antibacterial	[147]
Agastache honey	Australia	Phenol, 2,4-bis(1,1-dimethylethyl)	Antifungal	[148]
Aroeira honey	Brazil	Phenolics	Antifungal	[149]
Tupelo honey	United States	Sugars	Antimutagenic	[150]
Sage honey	United States	Sugars (lactulose, lactitol, and inulin)	Prebiotic effect	[83]
Buckwheat honey	United States	Sugars	Antibacterial	[147]
Safflower honey	China	Polyphenolics (protocatechuic acid, naringin, gallic acid, myricetin, and quercetin)	Anti-inflammatory	[151]
Sourwood honey	Malaysia	Phenolics, flavonoids and ascorbic acid	Antioxidant	[152]
Gelam honey	Malaysia	Amino acids (glycine, methionine, arginine, and proline)	Wound healing	[153]
Malicia honey	Brazil	Phenolics (procyanidins B1 and B2, epicatechin, and naringin)	Anxiolytic	[154]
Rhododendron honey	Turkey	Flavanones (hesperetin and luteolin)	Gastrointestinal protective	[155]
Manuka honey	New Zealand	Organic compound (methyl glyoxal)	Antibacterial properties	[156]
Red clover honey	Croatia	Volatile compounds (lilac aldehyde, phenylacetaldehyde, and benzaldehyde)	Anti-diabetic	[157]
Litchi honey	India	Protein (Major Royal Jelly Protein 1)	Anti-proliferative	[78]
Kanuka honey	New Zealand	Non-volatile compounds (syringic acid, 4-methoxyphenyllactic acid, and methyl syringate)	Anti-viral	[158]
Sesame honey	India	Phenolic compounds (rutin, apigenin, and quercetin)	Probiotic effects	[159]
Acacia honey	Malaysia	Flavones and flavanones (acacetin, chrysin, pinocembrin, and epicatechin)	Reduce adiposity and triglyceride levels	[160]

## 4. Clinical and Preclinical Evidence on Honey’s Mitigation of AC Toxicity

**Figure 4:**
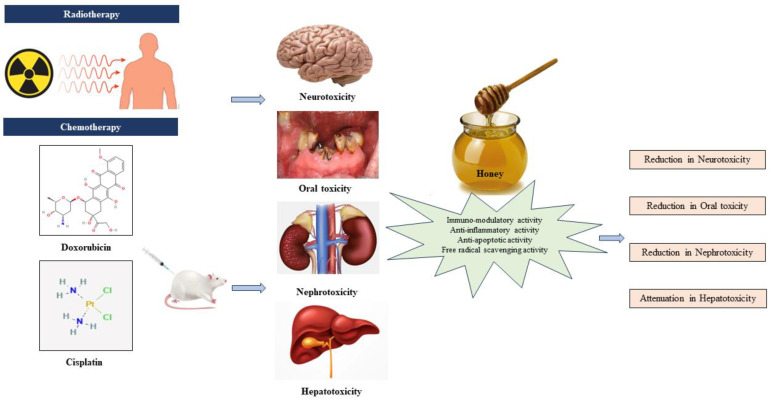
Honey and its therapeutic effect on anticancer chemotherapy-induced toxicity [22,23,24,25].

Preclinical evidence highlights the collaborative role of antioxidants, anti-inflammatory agents, immunomodulators, and anti-apoptotic agents in countering toxicity induced by AC drugs. Antioxidants neutralize free radicals, reducing oxidative stress and safeguarding healthy cells. Anti-inflammatory agents alleviate the inflammatory response, potentially mitigating adverse effects. Immunomodulatory agents boost the capacity of the immune system to identify and eradicate cancerous cells, while anti-apoptotic agents regulate cell death, preserving normal tissues. This collective synergy offers a promising approach to enhance chemotherapy efficacy and minimize side effects, paving the way for more tolerable and effective cancer treatments. Figure 4 shows the therapeutic effect of honey on AC-induced toxicity.

### 4.1. Antioxidant Mechanism of Mitigation

Overwhelming evidence indicates that AC is a trigger of ROS production in healthy or non-cancerous organs followed by the consequent emergence of oxidative stress. Foremost, AC agents such as cisplatin (CP), doxorubicin (DOX), cyclophosphamide (CYP), paclitaxel (PTX), docetaxel (DTX), and methotrexate (MTX) have been confirmed to induce ROS and oxidative stress [2,161,162,163]. During the aggressive attack of AC on the cell membrane as well as the mitochondria, ROS are generated, causing DNA damage and the degeneration of cells. Because redox imbalance is the gateway to numerous biological aberrations and pathologies, researchers have focused on targeting this process to prevent AC-induced ROS production with a number of natural products, including honey [12,164,165]. Honey’s multifaceted benefits extend to addressing oxidative stress induced by AC drugs because it contains phenolic acids, flavonoids, and other nutritional and non-nutritional bioactive compounds associated with antioxidant actions. In a rat model, hepatocellular carcinoma induced by diethylnitrosamine (50 mg/kg) and carbon tetrachloride (2 mg/kg) was treated with either CP, CYP, or 5-fluorouracil (5-FU) [12]. In the groups where honey was added, it was found that honey inhibits the hepatic oxidative stress via increasing the activities of antioxidant superoxide dismutase (SOD), catalase (CAT), glutathione peroxide (GPx), and glutathione-s-transferase (GST) and reducing the levels of glutathione (GSH). In this study, honey demonstrated antioxidant potential in countering the hepatotoxic side effects of CP, CYP, and 5-FU. Ibrahim et al. (2016) conducted a study to evaluate the effects of honey and royal jelly against sub-chronic cisplatin nephrotoxicity in rats. The results indicate that honey supplementation, especially when paired with cisplatin, mitigates oxidative renal damage and reduces levels of urea, creatinine, and uric acid in the serum [166]. The antioxidant effect of honey also exerts an improvement in histological lesions in the kidney. This suggests that honey may offer protection against CP-induced nephrotoxicity, providing a potential avenue to enhance the tolerability of this chemotherapy drug. Similar observations were reported, stating that honey administration enhances both liver and renal function through the detoxification of ROS [167]. The authors conclude that the protective effects could be associated with the antioxidant properties of honey. The antioxidant effects of Talh and Manuka honeys were evaluated in a rat model of CP-induced nephrotoxicity and hepatotoxicity [13]. The study revealed that both honeys depress hepatic oxidative stress, whereas there was no similar effect in the kidney, but only Manuka honey increased the activities of CAT. Although the two honeys improved renal and hepatic functions, only Manuka honey reversed the oxidative histopathological lesions in the kidney and liver, while Talh honey alleviated the histopathological changes in the liver. The study suggests that the antioxidant effect provided through the modulation of CAT, GPx, MDA, and GSH could be attributed to bioactive compounds such as octadecanoic acid, heneicosane, hydrocinnamic acid, kojic acid, pentacosane, hexadecanoic acid, and oleic acid present in the GC-MS profiles of Talh and Manuka honeys. The antioxidant action of natural honey protects against DOX-induced cardiac and hepatic damage in mice [168,169,170]. The cardiac and hepatic damage markers were markedly reduced in the group administered DOX + honey compared to the DOX group. According to the study, the protection of the organs from DOX toxicity is attributable to the content of polyphenolic compounds found in honey. MTX hepatotoxicity was also alleviated by honey-mediated increased activities of the antioxidant apparatus, including SOD, CAT, and GPx, in the liver. The reduction in oxidative stress by the antioxidant effect of honey also caused an improvement in liver function parameters and liver histology [11]. Similar findings were observed, where honey improved the total antioxidant capacity and markedly inhibited the production of the lipid peroxidation marker, malondialdehyde (MDA), against the MTX-induced side effect of intestinal toxicity [170]. Furthermore, Abdelhafiz et al. (2014) demonstrated honey’s antioxidant effect in attenuating CP-induced hepatotoxicity in male mice [171]. The study included groups treated with royal jelly, CP, and a combination of royal jelly and CP. Honey supplementation significantly improved the oxidative stress parameters and hepatic biochemical markers, indicating its potential in mitigating CP-induced liver damage. These findings highlight honey as a natural resource that may enhance the tolerability of chemotherapy by reducing AC-induced toxicities. In addition, the clinical study by Osama et al. (2017) corroborates the interesting findings from preclinical investigations. The study evaluated the antioxidant effect of honey and royal jelly in the protection of CP-induced acute kidney injury in patients with cancer [172]. The patients with cancer (32) were undergoing a cisplatin chemotherapeutic regimen consisting of 20 mg/m^2^ per dose for 4–5 days, known as a cisplatin cycle. The results show that the patients with cancer undergoing CP chemotherapy plus honey supplementation had lower serum levels of renal injury markers of creatinine and urea compared to those in the control group without honey supplementation. However, due to a small subject number, the authors recommend a further investigation using a larger sample size. This study suggests the antioxidant effect of honey on renal function, showing its beneficial role in patients with cancer. Studies are therefore suggesting that the integration of honey into AC protocols holds substantial promise in alleviating chemotherapy-induced oxidative stress.

### 4.2. Anti-Inflammatory Mechanism of Mitigation

The anti-inflammatory mechanism is integral in mitigating chemotherapy-induced toxicity, offering promise in enhancing the tolerability of cancer treatments. As chemotherapy triggers an inflammatory response with various side effects impacting patient well-being, anti-inflammatory agents become vital. They modulate key signaling pathways, inhibit pro-inflammatory cytokines, and alleviate oxidative stress, preserving healthy tissues. This approach is crucial in managing side effects like nausea and fatigue and optimizing chemotherapy’s effectiveness. A study by Hussein et al. (2013) confirmed Gelam honey’s anti-inflammatory properties via hindering NF-kB pathway activation in radiation-induced mucositis [173]. Oral mucositis is a worrisome side effect of AC [173]. It is an inflammatory response of mucosal epithelium to the cytotoxicity of AC and radiotherapy, leading to severe oral pain and ulceration, which may complicate the management of cancer [165]. A number of clinical trials have indicated the beneficial role of honey against AC mucositis [174]. The activation of NF-κB is the leading trigger for inflammatory response by translocation to the nucleus and the stimulation of gene expression for cytokines, including interleukin-1β (IL-1β), interleukin-6 (IL-6), and tumor necrosis faction-alpha (TNF-α). In addition, the studies by Rashad et al. (2010) and Yang et al. (2019) report honey’s role in mitigating mucositis severity during chemo-radiotherapy. The CP-induced activation of NF-κB and the consequent expression of inflammatory cyclooxygenase-2 (COX-2) in the kidney and liver was abrogated by honey according to the findings of Neamatallah et al. (2018) [13,175,176]. According to their study, honey supplementation inhibits oxidative stress and NF-κB activation. It is well known that NF-κB is an upstream regulator of pro-inflammatory mediators, including COX-2. The inhibition of NF-κB gene expression thus depresses the expression of hepatic and renal COX-2 and inflammation. Hamad et al. (2015) reported that pre-feeding rats with honey before exposure to CP alleviates inflammatory nephrotoxicity [14]. The anti-inflammatory effect of honey in this study was underscored by decreased cytokine and chemokine expressions and immune cell infiltration into the kidney compared to the CP-treated animals. The phosphorylation of NF-κB was also observed to be decreased due to honey feeding, leading to a significant decrease in the protein expression of NF-κB. These findings suggest that honey feeding could protect the kidney against CP nephrotoxicity via the suppression of inflammation and NF-kB activation. From enhancing IL-3 levels to mitigating the adverse effects of doxorubicin and cisplatin, honey emerges as a natural resource with the potential to enhance the tolerability and effectiveness of cancer treatment. Interleukin-3 (IL-3), a growth factor crucial for blood cell development, exhibits potential in fostering hematopoiesis and alleviating these unfavorable outcomes. In a study conducted by Kurniawan et al. (2020), an examination was undertaken to assess the influence of honey supplementation on IL-3 levels in breast cancer patients receiving AC [177]. The administration of honey (15 mL) thrice a day for 15 days demonstrated a noteworthy elevation in IL-3 levels within the honey-supplemented group in comparison to the control group. This suggests that honey supplementation could potentially enhance IL-3 levels, offering a means to counteract the blood cell count decline associated with AC. Hamad et al. (2015) demonstrated honey’s effectiveness in preventing CP-induced nephrotoxicity by reducing inflammation and oxidative stress [14]. The anti-inflammatory and antioxidant attributes of honey render it a potential complement in augmenting the well-being of individuals undergoing cancer therapy. As research continues, the multifaceted nature of honey positions it as a valuable asset in comprehensive cancer care strategies. These studies highlight honey’s diverse applications in cancer care and collectively underscore the importance of complementary approaches to AC, with natural products like honey offering multifaceted benefits in addressing the challenges associated with conventional AC.

### 4.3. Anti-Apoptotic Mechanism of Mitigation

The anti-apoptotic mechanism is pivotal for the mitigation of AC toxicity in healthy organ cells. The crosstalk of inflammation and apoptosis is well reported in drug-induced toxicity [178,179]. In a study conducted in 2022, Mohamed et al. (2022) illustrated the anti-apoptotic characteristics of honey in alleviating DOX-induced nephrotoxicity in male albino rats [180]. DOX elevated the renal expression of caspase-3, whereas the expression of Bcl-2, an anti-apoptotic protein, was reduced, suggesting the apoptotic effect of DOX in the renal tissue. The study found that apoptotic modulation is associated with the increased expression of poly (ADP-Ribose) polymerase-1 (PARP-1) in the DOX group.

PARP-1 is an enzyme marker for DNA damage which is well reported in AC toxicity, and it is a trigger of apoptosis in collaboration with oxidative stress-induced inflammation. Interestingly, the antiapoptotic effect of honey caused substantial reductions in caspase-3 and PARP-1 expression and an increase in Bcl-2 expression in the kidney. In another study, it was found that CP-induced oxidative stress contributes to the upregulation of apoptotic signaling via the activation of the NF-kB pathway [13]. The activation of NF-κB decreased Bcl-2 and increased the expressions of caspase-3 and Bax in the liver and kidney. These results were attributed to oxidative stress in addition to inflammation, leading to the apoptosis of cells. However, honey supplementation caused the deactivation of NF-κB and the mitigation of the DOX-induced apoptosis in both organs.

### 4.4. Immunomodulatory Mechanism of Mitigation

In a recent study led by Syam et al. (2021), the researchers delved into the effect of Dorsata honey (DH) as a supplementary approach to boost T lymphocytes in patients with breast cancer post-chemotherapy [181]. The group that received DH (n = 15), consuming 15 mL orally three times a day, displayed a noteworthy surge in T lymphocyte levels in comparison to the control group (n = 15) without honey. Blood samples taken on both day 0 and day 16 of chemotherapy unveiled heightened T lymphocyte levels. These findings suggest that DH might have a role in modulating the immune system and impeding the growth of tumor cells among patients with breast cancer undergoing AC. In a parallel study led by Kurniawan et al. (2020), the investigators explored the influence of honey supplementation on interleukin-3 (IL-3) levels in patients with breast cancer navigating chemotherapy [177]. The group receiving honey supplementation showed a significant elevation in IL-3 levels compared to the control group without honey. This hints at the potential of honey supplementation to amplify IL-3 levels, a hematopoietic growth factor acknowledged for its diverse contribution to blood cell formation. These observations point toward a conceivable supportive role for honey in mitigating the decline in blood cell counts associated with the side effects of AC. A study led by Zidan et al. (2006) showcased the effectiveness of Life-Mel honey (LMH) in averting neutropenia and lessening the reliance on colony-stimulating factors (CSFs) among individuals undergoing AC [182]. The severe side effect of febrile neutropenia often prompts the utilization of CSFs as both primary and secondary treatments for those experiencing grade 4 neutropenia. However, this approach can be financially burdensome and may bring about undesirable side effects. The incorporation of LM honey into the treatment regimen yielded promising results, with 40% of patients with grade 4 neutropenia treated with CSFs exhibiting neither recurrence nor further need for CSF treatment, ultimately enhancing their quality of life. The study concluded that providing LM honey to patients at an elevated risk of developing neutropenia due to AC mitigates the likelihood of pancytopenia and diminishes the necessity for CSFs. LMH, with its cost-effectiveness, lack of adverse effects, and straightforward administration, emerges as a viable and accessible intervention in addressing chemotherapy-induced neutropenia.

A recent comprehensive review and meta-analysis led by Hao et al. (2022) highlighted the effectiveness of honey in mitigating oral mucositis induced by radiation or chemotherapy (R/CIOM) among pediatric patients [183]. A meticulous exploration through electronic databases, such as Scopus, the Cochrane Library, PubMed, Web of Science, and Embase, was conducted to pinpoint relevant studies. The analysis included data from 316 patients, and their information was consolidated for the meta-analysis. The outcomes revealed a noteworthy reduction in recovery time among pediatric patients undergoing honey intervention, which was coupled with a significant decrease in the incidence of all grades of R/CIOM. These combined findings position honey as a promising and valuable complementary treatment for pediatric patients contending with AC-induced oral mucositis. AC oral mucositis (OM) poses a considerable challenge, particularly in pediatric patients with cancer who exhibit a higher prevalence of this condition compared to adults. Recent research by Zhang et al. (2022) delved into the potential of honey, renowned for its exceptional tissue-healing properties, in managing chemotherapy-induced OM in children [184]. Through the analysis of data from 346 children and adolescents with cancer, sourced from controlled trials, including both randomized and non-randomized studies, the findings indicate that honey could play a beneficial role in the treatment of OM in pediatric patients undergoing chemotherapy. The analysis demonstrated a significant improvement in the recovery time among pediatric patients treated with honey, showcasing its potential as a valuable intervention in managing OM in the pediatric population.

In the study conducted by Bhalchandra et al. (2018), the focus was on exploring the potential ameliorative effects of incorporating honey and royal jelly in male Wistar albino rats experiencing CP-induced changes in hematological parameters [167]. The rats were categorized into four groups: Group I, the control; Group II, which received cisplatin injections for 15 days; Group III, which was administered honey with royal jelly daily for the same period; and Group IV, which received both cisplatin injections and a daily combination of honey and royal jelly. Various parameters, including the Hb (hemoglobin) percentage, WBCs (White Blood Cells), RBCs (Red Blood Cells), platelets, and mean values of PCV (packed cell volume), MCV (mean corpuscular volume), and MCHC (mean corpuscular hemoglobin concentration) were meticulously evaluated. The CP-treated rats displayed a significant reduction in these parameters compared to the control group. Conversely, the group treated with royal jelly and honey exhibited a noteworthy increase in all blood parameters when compared to the control. Notably, the rats treated with dietary bee honey and royal jelly alongside CP demonstrated a substantial elevation in these parameters compared to those treated with cisplatin alone. These findings strongly suggest that the inclusion of honey and royal jelly in the diet serves as a natural preventive measure against CP-induced hematological alterations, potentially offering support during cancer treatment.

## 5. Conclusions

In summary, the investigation into honey as a remedy for AC toxicity unveils a promising avenue within AC treatment. While AC serves as a potent approach against malignant neoplasms, the consequential harm to healthy tissues, termed AC toxicity, necessitates the exploration of adjunctive solutions. However, it is not only the side effect of toxicity that complicates AC treatment, but also the development of chemoresistance. In this study, we emphasized the potential of honey for the mitigation of AC toxicities. Therefore, researchers, acknowledging the need for mitigating strategies, have turned their focus to natural compounds, with honey emerging as a compelling contender due to its diverse pharmacological activities. This comprehensive review intricately examined the interplay between honey and AC-induced toxicity, elucidating the underlying molecular mechanisms of mitigation. The multifaceted pharmacological activities of honey positions it as a versatile mitigator of the AC side effect of toxicity. The studies reviewed here show that oxidative stress, inflammation, and apoptosis are the frontline mechanisms of the AC side effect of toxicity. In parallel, the antioxidant, anti-inflammatory, and antiapoptotic effects of honey could mitigate AC toxicity. The findings presented here signify a ray of hope in the intricate management of the side effects of AC. The effect of honey on the development of chemoresistance, an important albatross to AC effectiveness, in vivo and in vitro remains to be reviewed. By harnessing the inherent properties of honey, researchers aim to contribute to a future where cancer treatments are not only robust in combating tumors but also considerate of the overall quality of life of patients both during and after chemotherapeutic treatment.

## Data Availability

No data were used for the research described in this article.

## Abbreviations

AC: Anticancer Chemotherapy; ROS: Reactive Oxygen Species; OHC: Outer Hair Cells; DNA: Deoxyribonucleic Acid; IL-3: Interleukin-3; ALT: Alanine Aminotransferase; OM: Oral Mucositis; DH: Dorsata Honey; LMH: Life-Mel Honey; CSFs: Colony-Stimulating Factors; R/CIOM: Radio/Chemotherapy-Induced Oral Mucositis.
